# An unusual association of hadrosaur and therizinosaur tracks within Late Cretaceous rocks of Denali National Park, Alaska

**DOI:** 10.1038/s41598-018-30110-8

**Published:** 2018-08-03

**Authors:** Anthony R. Fiorillo, Paul J. McCarthy, Yoshitsugu Kobayashi, Carla S. Tomsich, Ronald S. Tykoski, Yuong-Nam Lee, Tomonori Tanaka, Christopher R. Noto

**Affiliations:** 1 Perot Museum of Nature and Science, Department of Paleontology, Dallas, Texas 75201 United States; 2 University of Alaska, Department of Geosciences, Fairbanks, Alaska 99775 United States; 3 Hokkaido University Museum, Kita 10, Nishi 8, Kita-Ku, Sapporo, Hokkaido 060-0810 Japan; 4 School of Earth and Environmental Sciences, Seoul National University, Seoul, 08826 South Korea; 5 Department of Biological Sciences, University of Wisconsin–Parkside, Kenosha, Wisconsin 53141 United States

## Abstract

We report details of a unique association of hadrosaur and therizinosaur tracks found in the Late Cretaceous lower Cantwell Formation, Denali National Park, central Alaska Range, Alaska. This rock unit is now well-documented as a source of thousands of fossil footprints of vertebrates such as fishes, pterosaurs, and avialan and non-avialan dinosaurs. The lower Cantwell Formation in this area consists of numerous fining-upward successions of conglomerates and pebbly sandstones, cross-stratified and massive sandstones, interbedded sandstones and siltstones, organic-rich siltstones and shales, and rare, thin, bentonites, typically bounded by thin coal seams, and it contains a diverse fossil flora. We report the first North American co-occurrence of tracks attributable to hadrosaurs and therizinosaurs in the lower Cantwell Formation. Although previously un-reported in North America, this association of hadrosaur and therizinosaur tracks is more characteristic of the correlative Nemegt Formation in central Asia, perhaps suggesting that parameters defining the continental ecosystem of central Asia were also present in this part of Alaska during the Latest Cretaceous.

## Introduction

**Figure 1:**
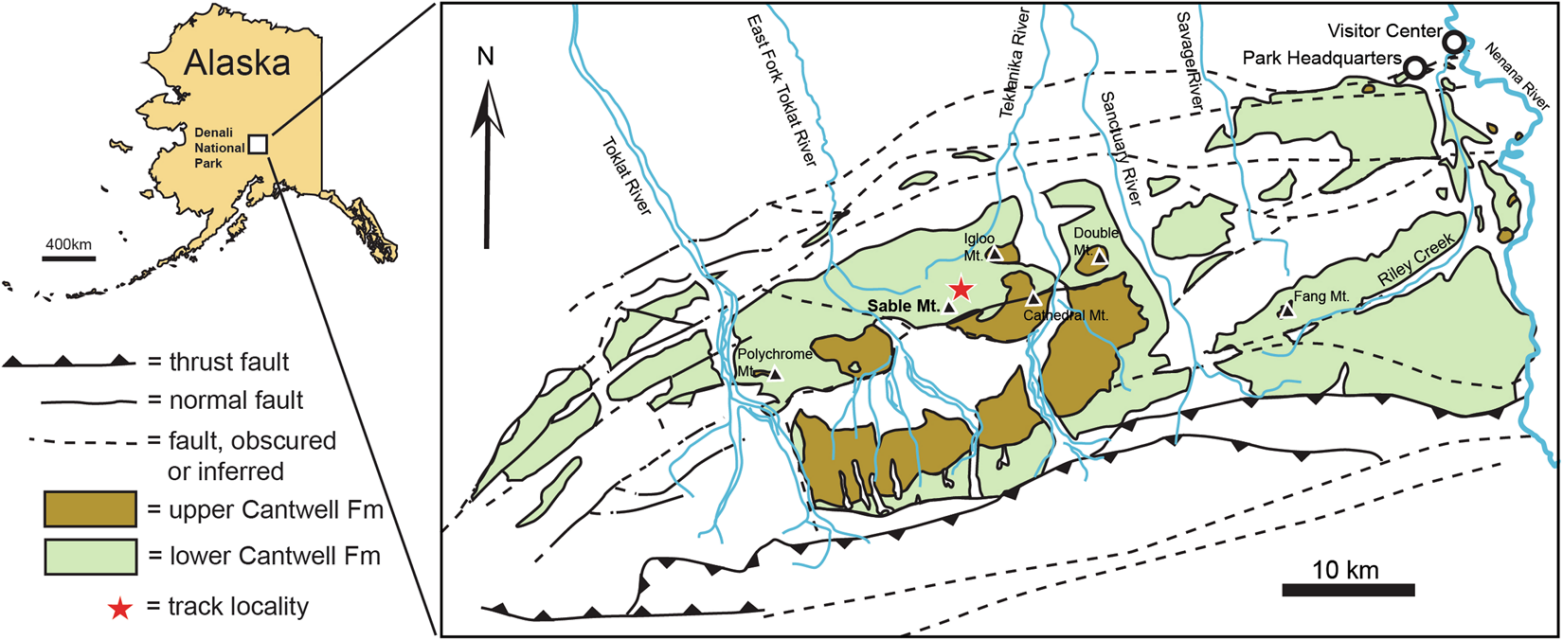
Map of Alaska and the Cantwell Formation within Denali National Park and Preserve. Star signifies study area. This map was drafted by R.S.T. in Adobe Illustrator, CC2017. (www.adobe.com/products/illustrator.html).

**Figure 2:**
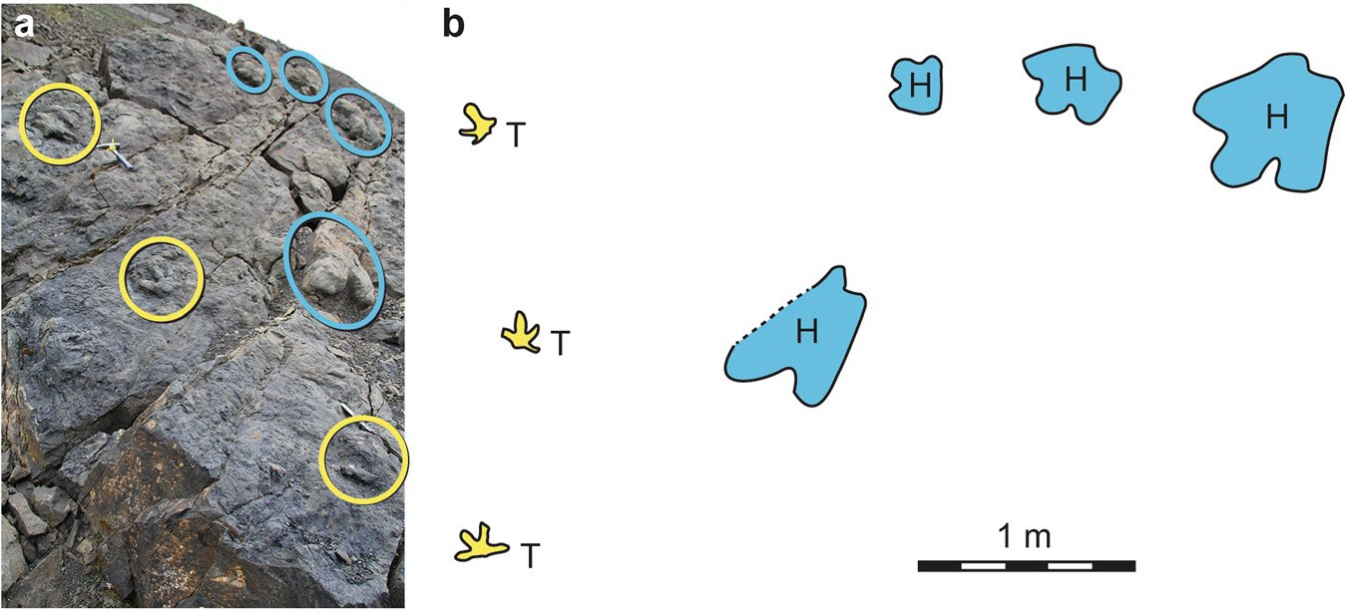
(**a**) Photo of large block in study area that demonstrates the co-occurrence of both hadrosaurid (in blue) and therizinosaurid (in yellow) tracks on the same bedding plane. (**b**) Line drawing of tracks on the slab in (**a**), with hadrosaurid tracks in blue, therizinosaurid tracks in yellow. Note the different sizes of hadrosaur tracks indicating multiple generations of this type of dinosaur. The oblique angle of Fig. 2 is due to the steep pitch of the block thus making an orthogonal view of the entire surface unavailable, though an orthogonal view of an isolated therizinosaur track has been previously illustrated^8^.

We report an association of hadrosaur and therizinosaur tracks unique to North America recently found in the Cretaceous lower Cantwell Formation, Denali National Park, central Alaska Range, Alaska (DENA, Fig. 1). This rock unit is well-documented as a source of thousands of fossil traces of vertebrates such as fishes, pterosaurs, and avialan and non-avialan dinosaurs^1–10^. Here we document the co-occurrence of tracks attributable to hadrosaurs (Ornithischia) and therizinosaurs (Saurischia) in the lower Cantwell Formation exposed in the Big Creek drainage of DENA. The tracks at this site are preserved in debris blocks of various sizes. Field observations confirm that the blocks eroded from a single sandstone layer farther up slope. Tracks preserved in the *in-situ* sandstone are only viewable in cross-section. While most specimens are single tracks of either hadrosaurs or therizinosaurs exposed on small blocks, one very large block confirms the tracks of both types of dinosaurs on the same bedding plane (Fig. 2). It is notable that this co-occurrence of therizinosaurs and hadrosaurids is, so far, limited to this single locality in the lower Cantwell Formation and this association is not documented elsewhere in North America. Rather, this co-occurrence is more characteristic of the dinosaurian fauna from correlative rocks in central Asia. Cretaceous Alaska represented the gateway for faunal exchange between these two continental landmasses, but previous studies focused on faunal exchange at the taxon-specific level^8,11–15^. This report presents new insights for the Cretaceous Beringian land bridge, suggesting that this association of dinosaurian taxa may be the result of paleoenvironmental parameters similarly present in the continental ecosystem of central Asia during the Latest Cretaceous.

## Results

### Geology and Paleoenvironment of the lower Cantwell Formation

The Cantwell Formation crops out extensively within DENA (Fig. 1). The lower Cantwell Formation comprises a stratigraphic thickness of up to 4000 m of terrestrial sedimentary deposits^16^ that rest unconformably upon lightly metamorphosed Jurassic to mid-Cretaceous marine sediments of the Kahiltna assemblage, and Devonian to Triassic strata of exotic terranes^9,17–22^. The lower Cantwell Formation is overlain, unconformably in places, by up to 3000 m thick Palaeocene volcanic to sub-volcanic and volcaniclastic deposits of the upper Cantwell Formation. The Cantwell Formation fills the Cantwell Basin, a 135-km long and up to 35-km wide, east-west-trending basin bracketed by the Hines Creek and McKinley faults, both major strands of the strike-slip Denali Fault system that bisects central Alaska (Fig. 1). The lower Cantwell Formation was assigned a late Campanian-early Maastrichtian age based on fossil pollen^19^. Recent zircon U-Pb dates from bentonites near Sable Mountain give ages of 71.5 +/− 0.9 and 71.0 +/− 1.1. Ma^9^. Another zircon U-Pb date from an outcrop along the East Fork of the Toklat River gives an age of 69.5 +/− 0.7 Ma^23^, placing the unit as partly correlative to other dinosaur-producing formations across Alaska. Paleotectonic reconstructions place the Cantwell Basin at a paleolatitude of 71° /−10°N, a high-latitude setting at the time of deposition^24^.

### Sedimentology at the therizinosaur track site

**Figure 3:**
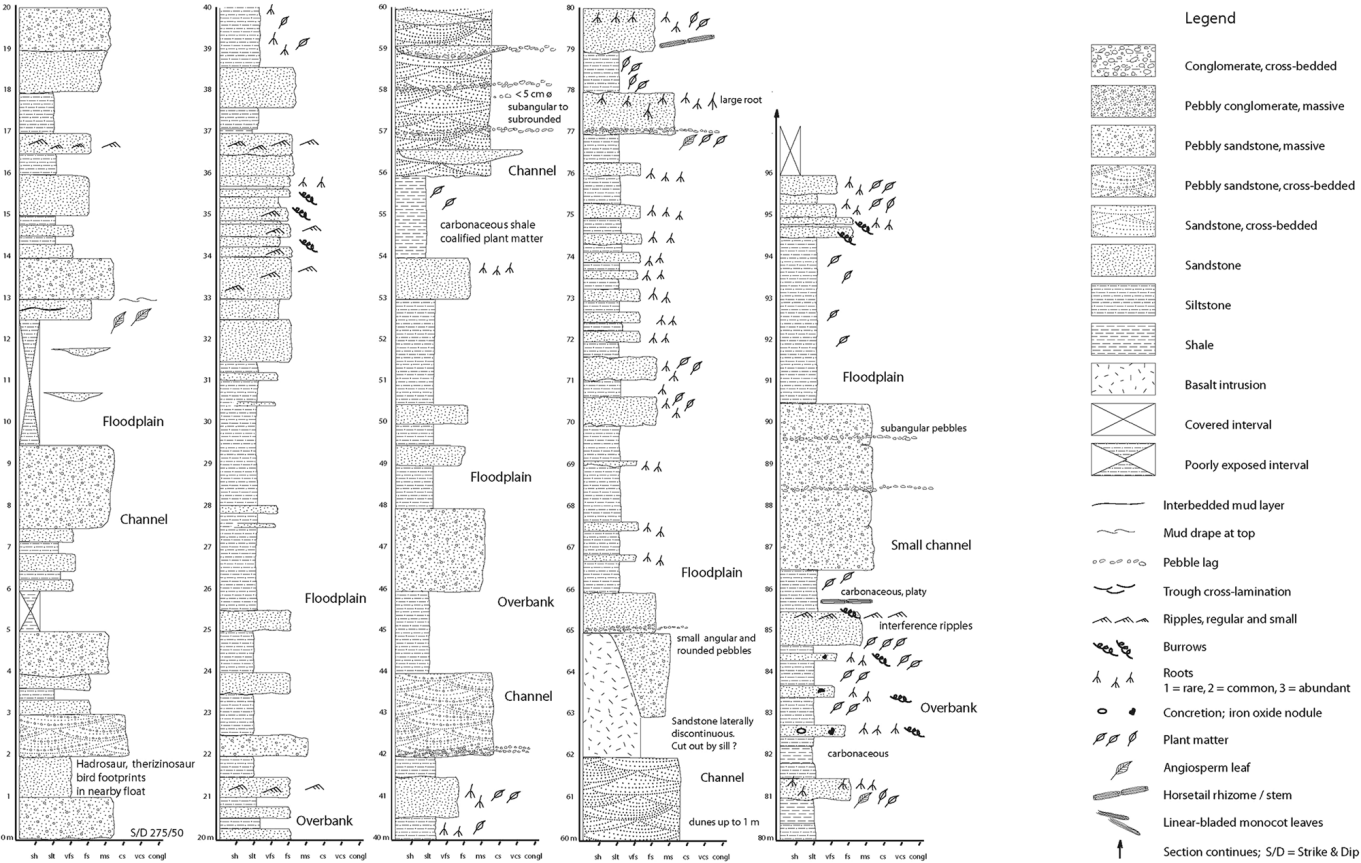
Measured stratigraphic section through the study area.

**Figure 4:**
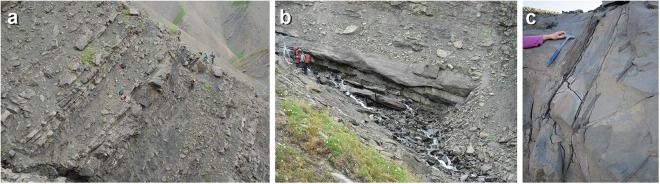
Outcrop photographs of lower Cantwell Formation in study area. (**a**) Representative exposure of rock unit, note the abundance of fine-grained strata. (**b**) An example of a splay channel within study area. (**c**) Fossil tree preserved within splay sand.

Rocks of the lower Cantwell Formation include conglomerates, organic-rich and fossiliferous sandstones, siltstones, mudstones and coals^2,19^ (Fig. 3). Lithologies around Sable Mountain consist of fining-upward successions of conglomerate and pebbly sandstones, cross-stratified and massive sandstones, interbedded sandstones and siltstones, organic-rich siltstones and shales, and rare bentonites, typically bounded by thin coals (Fig. 4). These rocks form a ~2000 m thick, fining-upward section representing distal alluvial fan and floodplain sediments^9^.

Coarsest-grained deposits constitute 2–10 m thick successions bounded by thick mudstone. Average maximum clast size is 12 cm. Medium- and fine-grained sandstones are present in both fining- and coarsening-upward cycles. These units (~10 to 50 m thick) consist of recurrent intervals of tabular fine- to medium - pebbly sandstones and mudstones. Well-stratified pebble conglomerates and sandstones are interpreted as traction-flow deposits onto gravelly channel floors and sand bars^25^. Horizontal gravel sheets represent gravel lags. Large-scale trough cross-beds (dunes) were deposited onto channel floors at the front or sides of migrating channel bars. Channelized conglomerate and pebbly sandstones are interpreted as vertically accreted channel and bar complexes characteristic of a predominantly sandy braided river^9^. Tabular, fining-upward, trough cross-bedded sandstones are interpreted as shallow distributary channels. Shallow channel-fills and tabular sandstones abruptly overlying siltstones and mudstones suggest a recurrent pattern of frequent avulsion and unconfined flow^26,27^. Mud drapes and desiccation cracks indicate rapid abandonment and periodic drying out of sediments as channels avulsed across distal fan lobes and adjacent basin marginal floodplains^22^.

Overbank deposits include laterally extensive, thin, fine- to very fine-grained sandstones interbedded with siltstones, mudstones and coaly shales (Fig. 4a). Some sandstones are present in coarsening-upward cycles, with ripple cross-lamination and rhizoliths at the top. Thin tabular sandstones encased in mudstones are also present. Interbedded fine-grained sandstones and siltstones may be heavily burrowed and rooted. Fine-grained sandstones and mudstones contain tree trunk impressions, root and branch casts, and partly coalified fossilized wood up to 40 cm long and 30 cm wide. Iron oxide nodules and concretions may occur.

Coarsening-upward, cross-bedded sandstones represent crevasse channels or crevasse deltas that filled shallow floodplain lakes or ponds. More heavily burrowed and rooted, thin sandstones and siltstones interbedded with mudstones probably represent thin crevasse splays deposited further from active distributaries. Laterally continuous, tabular sandstones are interpreted as sheet flood deposits. Similar units from other parts of the Cantwell basin are interpreted as extensive unconfined sheet flood deposits based on lateral continuity, normal grading, limited thickness and a tabular, non-channelized morphology^19^. Coaly shales and laminated shales represent backswamp deposits and small ponds or lakes in distal fan settings away from active fan lobes or on adjacent floodplains (Fig. 4b). Rooted sandstones and mudstones represent weakly developed paleosols which, in addition to the occurrence of buried upright trees (Fig. 4c), suggest high rates of aggradation on unstable landscapes.

### Paleobotany at Sable Mountain

Paleoflora from the lower Cantwell Formation has been studied in detail at two localities: Sable Mountain and upper Riley Creek^7,9,28^. Sample batches at both localities are parautochthonous and it is unlikely that they represent *in situ* vegetation. Monotypic plant fossil assemblages, however, probably indicate plants that were growing on site, and were preserved in low-energy environments such as lacustrine and backwater settings. At Sable Mountain, the lower Cantwell contains a diverse flora consisting of horsetails, ferns (e.g., *Asplenium* sp. and *Cladophlebis* sp.), shoots, cones and seeds of the deciduous conifers Metasequoia sp., (*M. occidentalis?)*, *Parataxodium* sp. *(P. wigginsii?)*, Glyptostrobus sp., Tumion gracilis, Ce*phalotaxopsis heterophylla*, and *Larix sp*. Angiosperm leaves and conifer shoots often form leaf mats pointing to synchronous leaf loss and a deciduous habit. Angiosperms are represented by a relatively diverse assemblage of broad-leaved and linear-bladed monocotyledonous and woody dicotyledonous leaf forms. Dicot leaf fossils are of menispermoid, trochodendroid, platanoid and higher hamamelid affinities. Palmately veined menispermoid (e.g., *Menispermites septentrionalis*) and trochodendroid leaves (e.g., *Trochodendroides richardsonii*), and leaves of betulaceous form (e.g. Corylites sp. and an Alnus-like leaf) are most abundant and typically occur in fine-grained floodplain environments in association with *Metasequoia* sp. and possibly *Parataxodium* sp. Platanoid types, such as *Platanites* sp. and *Pseudoprotophyllum borealis*, are more common in coarser-grained sediments, suggesting a taphonomic bias or it may indicate a preference for riparian environments or more proximal alluvial fan settings^7,28^.

Other slumped blocks incorporate large pieces of fossilized and partly coalified conifer wood, leafy shoots of taxodioid-cupressaceous conifers, rhizome and stem segments and tuberli of *Equisetum arcticum*, and fern frond fossils. This implies the growth site may be a semi-open moist habitat in a groundwater-fed or frequently inundated low-lying floodplain that retained water in shallow lakes, backwater ponds, marshes, and abandoned channels. In contrast, reed-like monocot leaf impression and *Equisetum* sp. fossils associated with finer-grained fluvial or overbank sands and muds may extend the habitat interpretation to a riparian setting^29^. As drainage improved over time, wet sandy soils became more amendable for other plants to succeed. Sandstone with large-sized root horizons indicates periods of non-deposition between episodic high discharge events. The dark color of the finer-grained facies, sparse burrowing, coalification of wood, concretions, and iron oxide nodules suggest water tables remained close to the surface, while shallow lakes, ponds, and abandoned channels accumulated fine-grained sediments and supported a shallow water aquatic plant and lake margin forest community^9,26^.

**Figure 5:**
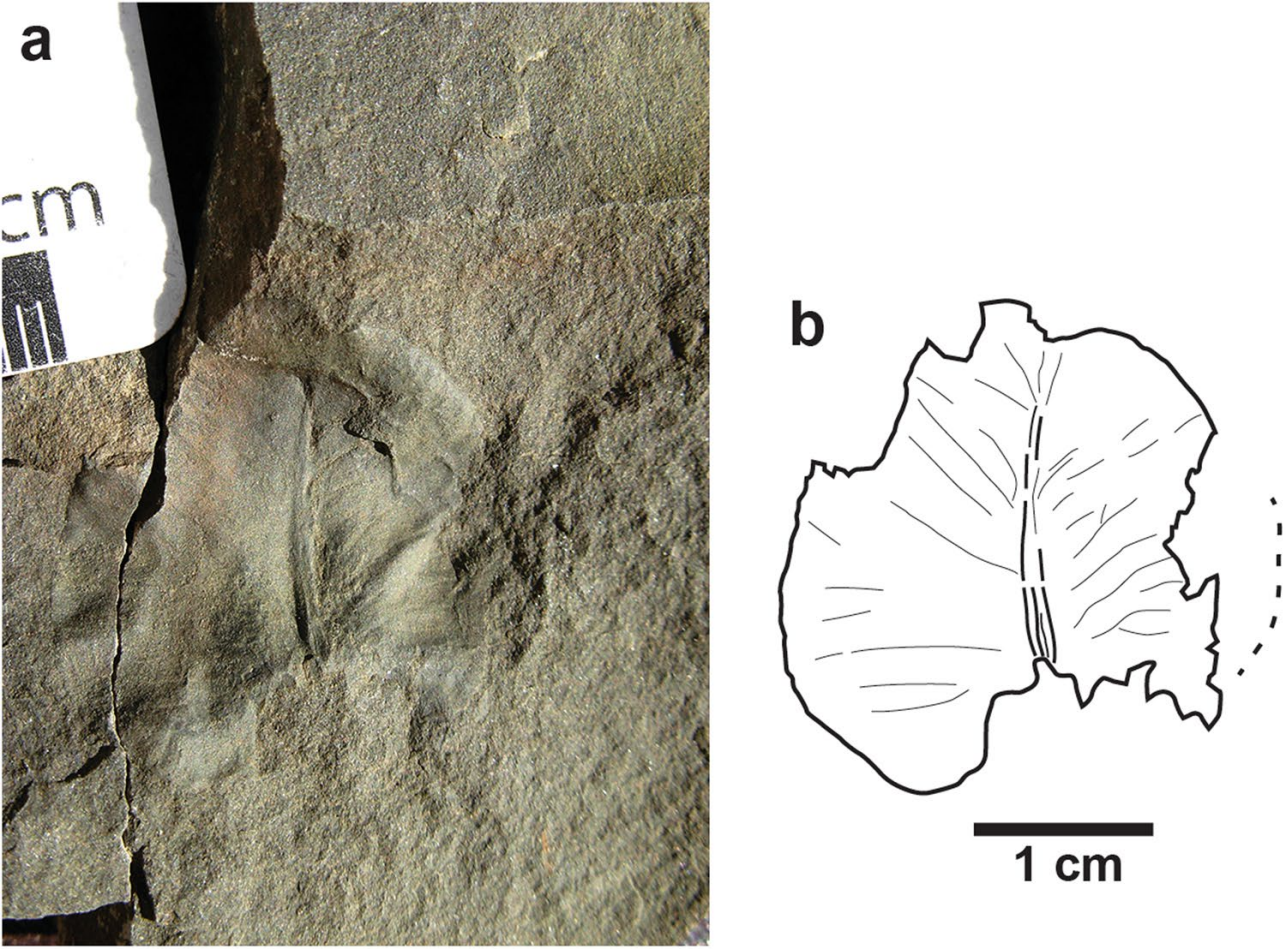
Photograph (**a**) and line drawing (**b**) of nymphaceous leaf found in study area. This plant is indicative of standing water.

A small fossil leaf with a deeply cordate base, entire margin, a broad and tapered multistrand midvein and pinnate-brochidodromous venation is found at Sable Mountain in massive fine-grained sandstone that preserved the hadrosaur and therizinosaur tracks (Fig. 5). The leaf morphology and distinctive venation suggest a basal lineage such as Amborellales and its earliest sister group, the Nymphaeales, closely resembling leaves of extant *Nuphar* sp. (water lily). The Nymphaeaceae may date to as early as the Early Cretaceous, and leaves resembling extant *Nuphar* sp. have been named from Early Late Cretaceous outcrops along the Yukon-Koyukuk Rivers, and Paleocene of the Rocky Mountains^30–32^. The Nymphaeaceae are an aquatic plant group characterized by thin floating leaves that grow across still water ponds and shallow lakes, and sedimentary data indicate that the aquatic growth habit may be ancestral to this plant group^33^. The low-relief of the leaf fossil impression indicates that in life the specimen was thin-bladed. The relative integrity of this delicate leaf indicates it was not transported far, and transport by streams can be excluded. Most likely, this leaf floated on the surface of a quiet, shallow water body distal from main channels. Our taphonomic interpretation suggests the leaf was encased in rapidly settling overbank sediment while still suspended in water. This water-lily-like leaf fossil supports the assumption that the hadrosaur and therizinosaur tracks at Sable Mountain were made in shallow standing water prior to deposition of a crevasse-sand body that was deposited into a shallow pond or lake, preserving them.

### Depositional Environment Summary

Paleobotanical evidence indicates wetland and possibly riparian plant communities in the area. The study area was either a distal wet alluvial fan on a low-gradient slope, or an interfan and/or fan fringe interfingering with an adjacent axial valley floodplain^34^. Mudstones were deposited outside of the main active depositional lobe or in abandoned channels. Deposition from unconfined overbank flow occurred when channels shifted or where floodwaters overtopped banks while passing through a network of smaller, sediment-choked, distributary channels. Sheet-flow is common on alluvial fans, involving an entire active lobe and becoming unconfined across the distal fan and on the adjacent alluvial plain^35^.

### Paleoclimate of the lower Cantwell Formation

Paleoclimate during deposition of the lower Cantwell Formation was reconstructed from CLAMP (Climate Leaf Analysis Multivariate Program^36–39^) analysis of leaf material and stable isotopic analysis of organic matter and wood^9,23,28^. CLAMP results for the Maastrichtian lower Cantwell Formation at Sable Mountain indicate a mean annual temperature of 7.4 +/− 2.4 °C, a warmest month mean temperature of 17.1 +/− 3.2 °C, and a coldest month mean temperature of −2.3 +/− 3.8 °C. CLAMP results also indicate growing season precipitation of 229.4 +/− 671.8 mm, three wettest months precipitation of 176.5 +/− 280.2 mm, three driest months precipitation of 141 +/− 186 mm and a growing season that was 4.8 +/− 1.4 months long. The relative humidity, calculated from CLAMP was 73.9 +/− 7.4%^40^.

Carbon stable isotope (δ^13^C) data were determined for bulk organic matter and wood fragments from the lower Cantwell Formation at the East Fork of the Toklat River at a site ~9 km west of the study area. Bulk organic matter values were used to determine values of δ^13^C_atm_, and then these values and values for δ^13^C_wood_ were used to determine isotopic fractionation of C_3_ plants during carbon assimilation^41^. This calculated value was then substituted into an equation^42^ to determine mean annual precipitation (MAP) for the lower Cantwell Formation. Sampling at the East Fork of the Toklat River surrounded a bentonite with a radiometric age of 69.5 +/− 0.7 Ma^23^, so MAP values were calculated for the Middle-Maastrichtian Event (MME), an intense greenhouse episode at ~ 69 Ma^43^, as well as for pre- and post-MME time. Calculated MAP estimates range from 353–1050 mm/yr before the MME, 168–470 mm/yr during the MME, and 475–1451 mm/yr after the MME^23^. Calculated temperature values are consistent with other Maastrichtian high-latitude paleobotanical temperature estimates, but paleoprecipitation values are lower than those reported from near-coastal high-latitude Maastrichtian environments elsewhere^28,44–46^.

### Tracks and track makers

The dinosaur tracks of interest were discovered on debris blocks of sandstone, high on a remote mountain slope in the Big Creek drainage on Sable Mountain, in DENA (Fig. 2a). The siltstone and fine-grained sandstone blocks tumbled or slid downslope to their current position, but can be traced confidently to their source horizon higher on the mountain. The lower Cantwell Formation is the only rock unit comprising the mountain. The tracks are raised-relief features, formed as sediment in-fillings of footprint impressions in softer, underlying sediment. Subsequent erosion of the softer sediment exposed the tracks as they are now. While smaller blocks preserve individual tracks, one large block preserved two different kinds of dinosaur in association (Fig. 2).

#### Hadrosaurid tracks

**Figure 6:**
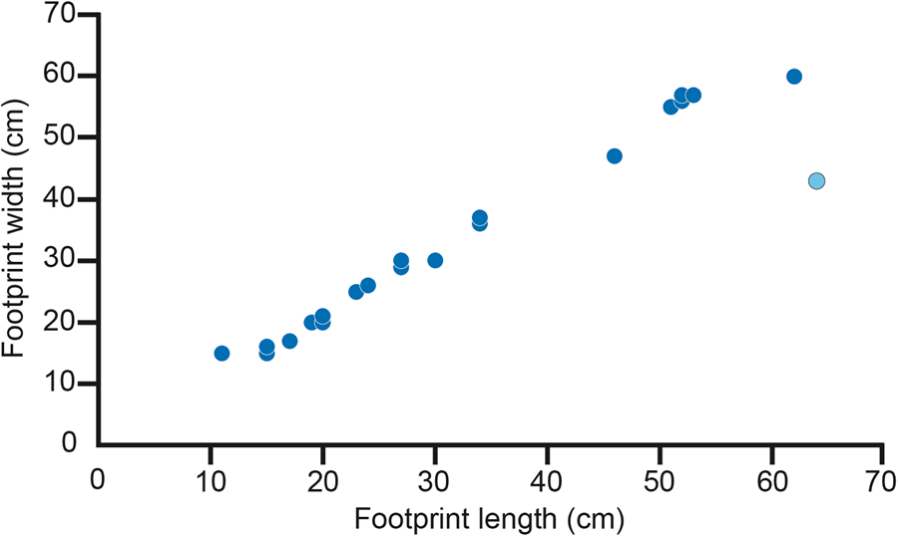
Graph of length and width measurements of hadrosaurid tracks from study area showing a linear relationship in size from small to large. Such a relationship indicates the presence of a multi-generational herd of these dinosaurs in this area.

The most abundant tracks at the site are attributable to hadrosaurid dinosaurs (Fig. 2b). Except for a paired set of manus-pes impressions attributable to a very young hadrosaur^10^, only pes impressions were found. The pes tracks are tridactyl, wider than long, with digits that are short, wide, and terminate bluntly. They also have wide, bi-lobed posterior margins that are wider than the proximal part of the impression of digit III. Tracks range in size to include those made by adults, subadults, and juveniles (Fig. 6). These morphological features are consistent with tracks that have been attributable to hadrosaurids and are referred to *Hadrosauropodus* isp^47–49^. Morphologically similar tracks are reported elsewhere in the lower Cantwell Formation of Denali National Park^4^.

#### Therizinosaurid tracks

The first recognized Alaskan therizinosaur track was described from a single four-toed track at this site and attributed to the ichnogenus *Saurexallopus*^8^. The track was considered that of a therizinosaur because of the presence of four slender weight-bearing digits. Members of at least two clades of theropods are known to have four forward-facing toes on the pes, therizinosaurs and the closely related oviraptorosaurs, particularly oviraptorids. While there are a growing number reports of *Sauroexallopus*^50,51^, there are differing opinions on the attribution of this ichnogenus^8,50,51^.

**Figure 7:**
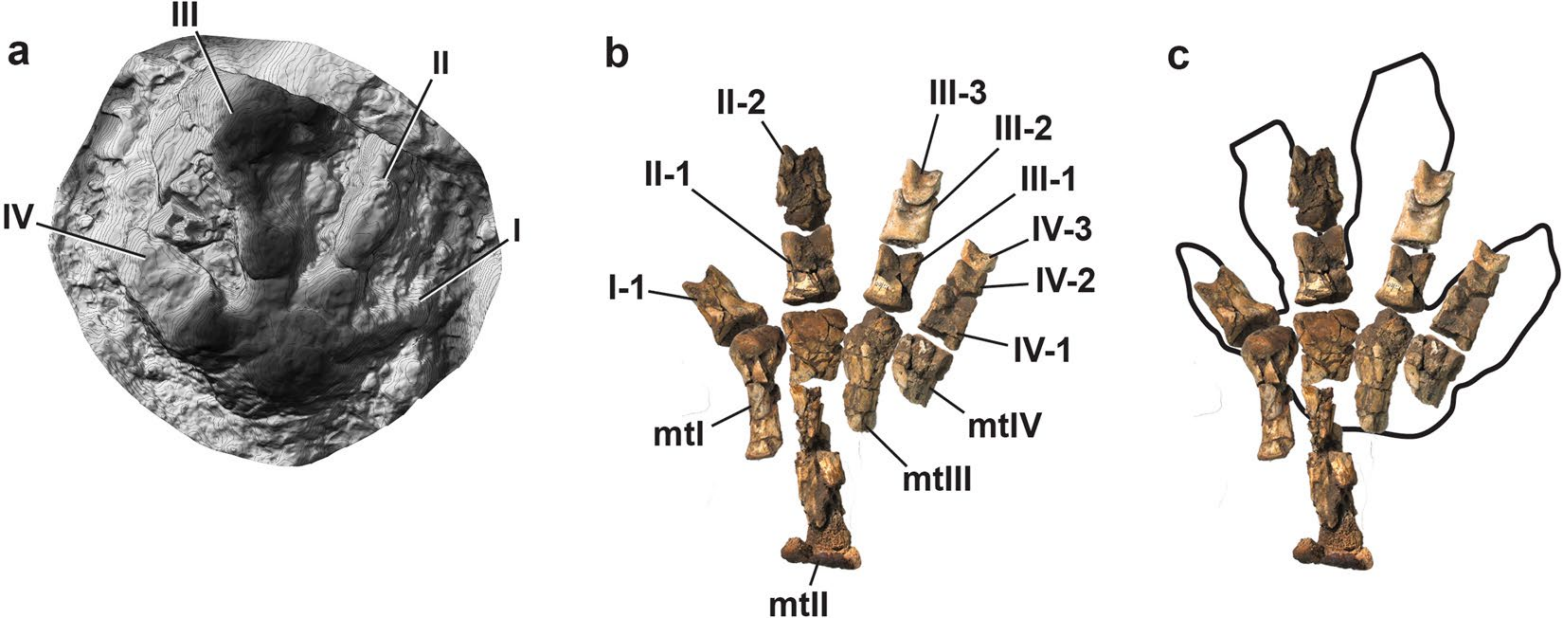
Comparison of a digital map of a therizinosaurid track (DMNH 2010-07-01) (**a**) from study area with the articulated foot of the therizinosaurid *Erlikosaurus andrewsi* (**b**). The similarities in digit proportions are clear in (**c**), an overlay of illustration b on a line drawing of illustration a. Because the track shown in a is preserved in raised relief, the line drawing has been inverted to correspond to the order of digits if preserved in negative relief.

**Figure 8:**
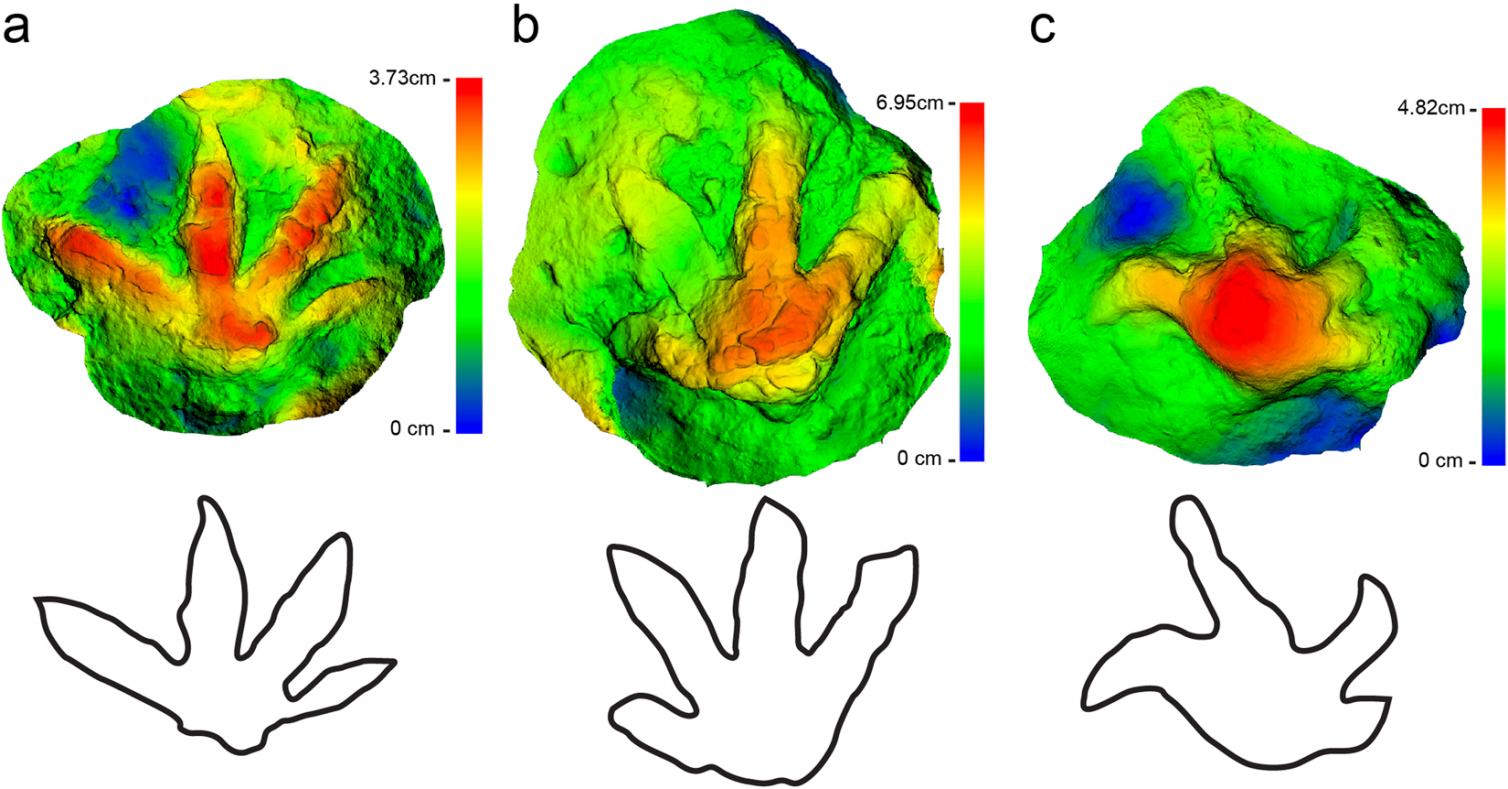
Digital elevation maps of three additional tracks from study area that illustrate the variation in the morphology of these therizinosaurid tracks. (**a**) DMNH 2013-08-04; (**b**) DMNH 2013-08-06; (**c**) DMNH 2014-11-05.

**Figure 9:**
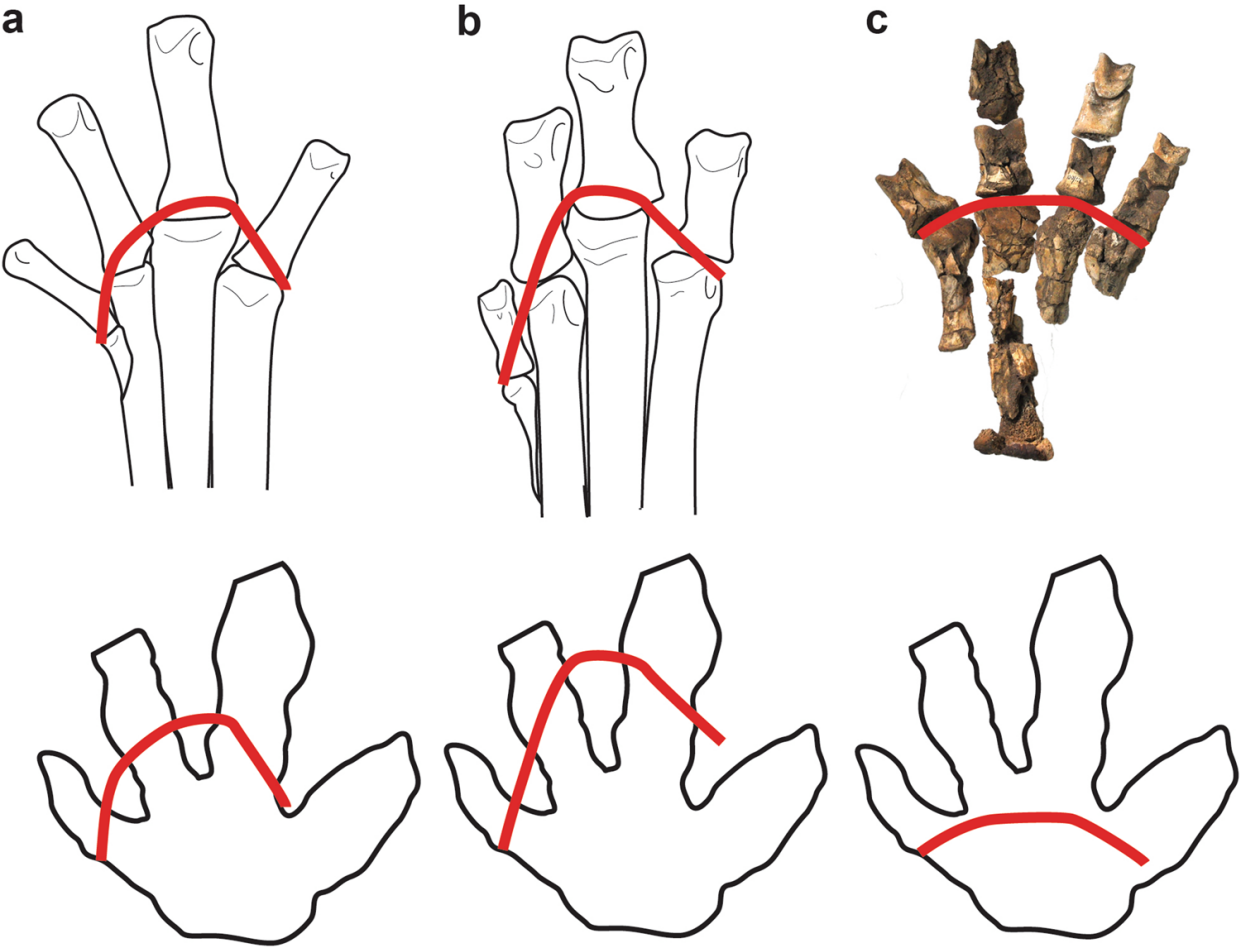
Comparison of foot skeleton in oviraptorosaurs and derived therizinosaurid. (**a**), line drawing of CMN 8538 *Chirostenotes pergracilis*; (**b**), IGM 100/973, *Khaan mckennai*; (**c**) IGM 100/111, *Erlicosaurus andrewsi*. Red arcs drawn across distal metatarsal surfaces. Lower outlines are of Big Creek track DMNH 2010-07-01. Same red arcs superimposed on track outline. Notice good fit of arc from ‘c’, compared to ‘a’ and ‘b’.

**Figure 10:**
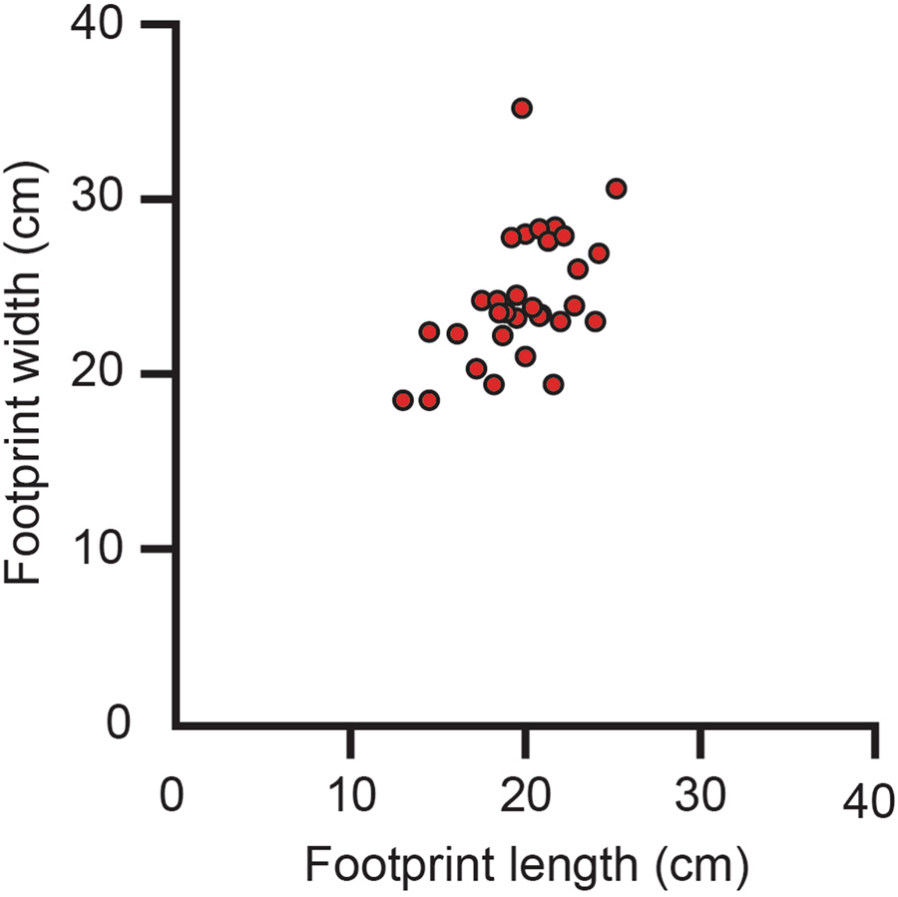
Graph of length and width measurements of therizinosaurid tracks from study area showing a cluster rather than a clearly defined segregation of size. This distribution suggests a much more restricted age group than what is observed in the population of hadrosaurid track measurements^84,85^.

The Big Creek site has now produced at least 31 tetradactyl footprints among the more numerous hadrosaurid examples on the locality (Figs 2, 7, 8, Fig. S1, Table S1). At least three of the tetradactyl footprints appear to constitute a trackway made by a single individual on the same bedding plane as several *Hadrosauropodus* tracks made by at least three individuals (Fig. 2). These tetradactyl footprints average 21.4 cm in length. Using the standard equation to determine hip height from footprints of dinosaurs as approximately four times the track length^52,53^, these pes tracks suggest a hip height estimate of approximately 85.6 cm for this trackmaker. Unlike the pedal skeletal proportions of the caenagnthathid *Chirostenotes pergracilis* and the oviraptorid *Khaan mckennai* (Fig. 9a,b), the skeletal structure of a therizinosaurid foot is a close match to the morphology of tetradactyl tracks from the site (Figs 7, 9c). The broad, shallow arc of the distal metatarsal condyles of therizinosaurids are a better fit for the Big Creek tetradactyl tracks than the narrower and deeper arc drawn across the four distal metatarsal condyles of the proximally-positioned metatarsal I to the distally protruding metatarsal III of oviraptorosaurian taxa (Fig. 9). Given the robust proportions of pedal digit I and the resulting presence of four weight-bearing digit impressions, coupled with retention of pointed, claw-like terminations of the digits, we attribute these tetradactyl tracks to therizinosaurids. The tracks range in size (Fig. 10; Table S1), though not as widely as the hadrosaurid tracks from the site.

Were these tetradactyl tracks made by a different theropod taxon, such as an oviraptorosaur? Unlike most other non-avian theropods, pedal digit I of oviraptorosaurs is positioned medial to the shaft of metatarsal II, but retains the relatively small proportions seen in most other non-avian theropod taxa^54–57^ (Fig. 9a,b). Also, pedal digit I most likely flexed in the same plane and direction as the toes of digits II-IV^58^. The size disparity between pedal digit I and the other pedal digits is seen in articulated specimens of the North American caenagnathid *Chirostenotes pergracilis* (CMN 8538) and the Asian oviraptorid *Khaan mckennai*, (Fig. 9a,b). In both oviraptorosaurs the articulation between metatarsal I and phalanx I-1 is positioned well proximal to the distal ends of metatarsal II, III, and IV (Fig. 9a,b). The distal end of phalanx I-1 does not reach the mid-shaft of phalanx II-1 in *Chirostenotes* (Fig. 9a), and the distal tip of phalanx I-2 does not reach the distal end of the adjacent phalanx II-1^50^. In *Khaan*, phalanx I-1 barely reaches the articulation between metatarsal II and phalanx II-1 (Fig. 9b) and the distal tip of phalanx I-2 only reaches about the mid-point of the adjacent phalanx II-1^57^. Indeed, the distal tip of digit I barely extends beyond the plane through the articulation between metatarsal III and phalanx III-1 in *Khaan*^57^. An oviraptorid pedal digit I contacting the substrate would create only a small toe impression compared to digits II through IV. A digit I impression left by the caenagnathid *Chirostenotes* would be only slightly longer, but would retain a narrow, delicate profile compared to impressions of digits II through IV.

In contrast, the feet of derived Late Cretaceous Therizinosauridae have four large weight-bearing toes^52,53,59^. Therizinosaurs show a unique evolutionary history in foot structure. Basal, Early Cretaceous therizinosaurs such as *Falcarius utahensis* have four slender toes and retain a functionally tridactyl foot (weight-bearing digits II-IV) with a small first digit like most non-avian theropod dinosaurs^60^. Derived therizinosaurs, the Therizinosauridae, have greatly modified foot structures. Therizinosaurids such as *Segnosaurus galbienensis* and *Therizinosaurus cheloniformis* have a functionally tetradactyl foot^61^ with digit I as robust as digits II-IV. The pedal digits are almost equally robust, and pedal phalanges are much wider than high, unlike in basal therizinosaurs and other theropods. The distal end of metatarsal I diverges medially in *Nothronychus graffami*, *Neimongosaurus yangi*, *Segnosaurus galbienensis*, and *Therizinosaurus cheloniformis* and is medially rotated in distal view. Because of its divergence and rotation, the angle between digits I and II is wider than that in basal therizinosaurs. Taken together, derived therizinosaurids from Asia (*Neimongosaurus yangi*, *Segnosaurus galbienensis*, and *Therizinosaurus cheloniformis*) and North America (*Nothronychus grafammi and Nothronychus mckinleyi*) have a unique combination of pedal features (tetradactyl foot, robust digit I, wide phalanges, wide angle between digits I and II), which is different from basal therizinosaurs and other non-avian dinosaurs^52,60,62,63^. Therefore, the tracks found in this study site are consistent with the known skeletal anatomy of therizinosaurs.

## Discussion

The association of hadrosaurid and therizinosaurid tracks in Late Cretaceous sediments of south-central Alaska/Beringia is noteworthy because it is not reported elsewhere in North America. Rather, this faunal association is more characteristic of correlative rocks in central Asia^64^. It is particularly intriguing given that the hadrosaurids likely had a complex social dynamic^5^, thereby prompting hypotheses of scenarios to explain this rare co-occurrence of taxa. The first scenario is that although the tracks are spatially connected, the taxa were separated temporally. In other words, a herd of hadrosaurids and the therizinosaurids walked across this landscape at different times, and did not encounter each another in doing so. Alternatively, this co-occurrence was biologically linked, and these taxa co-existed when and where conditions were right. Such a scenario warrants some speculation about the paleobiological significance of this co-occurrence of fossil taxa. Extant taxa provide clues to the possible interaction, because different species of extant taxa can co-exist for beneficial reasons, such as improving resource acquisition or reducing predation risks.

The co-existence of African zebras and wildebeests has received a great deal of scientific attention, though it remains to be fully understood. Traditional views stemming from studies of the 1960s and 1970s suggested that interspecific competition was a dominant influence that shaped this co-existence^65,66^, but further observation and study suggested instead that predator avoidance played as significant a role^66^. It is now recognized that predator avoidance is complex, as enhanced sensory perception of one herbivore species verses another can offer benefits for co-existence^66,67^. Landscape heterogeneity can also play a role and can contribute specifically to the dynamics in the case of wildebeests and zebras^68^ mentioned above.

Recent work on jaw mechanics and endocranial anatomy of therizinosaurs allows for comparison of some attributes between this clade and the better-known hadrosaurs. A study of evolutionary trends in jaw mechanics of therizinosaurs noticed a change in bite force through time^69^. More derived Late Cretaceous therizinosaurids such as *Erlikosaurus andrewsi* and *Segnosaurus galbinensis* had a reduced bite force compared to earlier therizinosaurs such as *Falcarius utahensis*^69^. Another examination that modeled the bite force in *Erlikosaurus andrewsi* suggested that the bite force in the hadrosaur *Edmontosaurus* was greater than that for *Erlikosaurus*^70^. The lesser bite force for *Erlikosaurus* better served in stripping and cropping leaves, rather than active mastication^70^. While *Erlikosaurus* and *Segnosaurus* are from rocks older than the lower Cantwell Formation, they are the only derived therizinosaurids with described jaws within the clade that includes *Therizinosaurus*, the therizinosaurid correlative with this Alaskan rock unit^15^. Given this evolutionary relationship, it is reasonable to speculate that *Therizinosaurus* had a similarly lesser bit force.

Hadrosaur tooth microwear, specifically in *Edmontosaurus*, suggested hadrosaurs were actively chewing grazers rather than browsers^71^. It is tempting to conclude that these two kinds of herbivorous dinosaurs co-existed in a mixed-taxonomic herd because of reduced interspecific competition for food resources. However, this ecological process has fallen out of favor among workers studying modern ecosystem processes and interactions^66,68^, so caution must be exercised before uncritically applying this phenomenon to an example from the fossil record.

Although the sample size of fossil specimens is limited, it seems that therizinosaurs may have had well-developed senses of olfaction and hearing^72^. Such sensory attributes almost assuredly served an important role in activities such as foraging, predator evasion, and/or social complexity^72^. In contrast, while increased olfaction was proposed for at least some members of the Lambeosaurinae^73^ a reevaluation of the nasal cavity in this group of hadrosaurs suggested instead that the nasal region of hadrosaurs was conservative^74^. Again, it is tempting to consider the differences in sensory adaptations and capabilities in these taxa that might have served a role as a mutually beneficial predatory avoidance mechanism for the more inclusive herd. However, if co-existence for predator avoidance was the reason for this co-occurrence of taxa and their tracks, then one would expect that the demographic profile of the two taxa to be similar, as expressed by the length-width measurements of the tracks for each taxon. Measurements of the tracks at the site do not plot to the same sort of herd demographic pattern (Figs 6, 10). Given the similarity of track preservation, it seems likely that these two taxa occupied the same environment at the same time. It would appear though that the taxa were not linked by complex mutually beneficial behavior. Rather it is more likely that an aspect of the environment led to the co-occurrence of these taxa and their tracks.

The correlative Nemegt Formation of Mongolia is thought to have been deposited under more humid conditions than either the older Cretaceous Djadokhta or Baruungoyot formations in the same area^75,76^. The Nemegt Formation has produced a diverse terrestrial vertebrate fauna, including skeletons of hadrosaurids (*Saurolophus angustirostris*) and therizinosaurids (*Therizinosaurus chelonifomis*), and there are footprint localities dominated by hadrosaurid tracks^47,64,77^. To account for the stratigraphic distribution of hadrosauroids in the Mongolian sequence of sediments, it was offered that the presence or absence of hadrosauroids in the Late Cretaceous rock units of Mongolia may be linked to the climatic conditions under which each rock unit was deposited^76^. More specifically a shift from more arid to more mesic conditions may account for the immigration of derived hadrosaurids from North America^76^.

**Figure 11:**
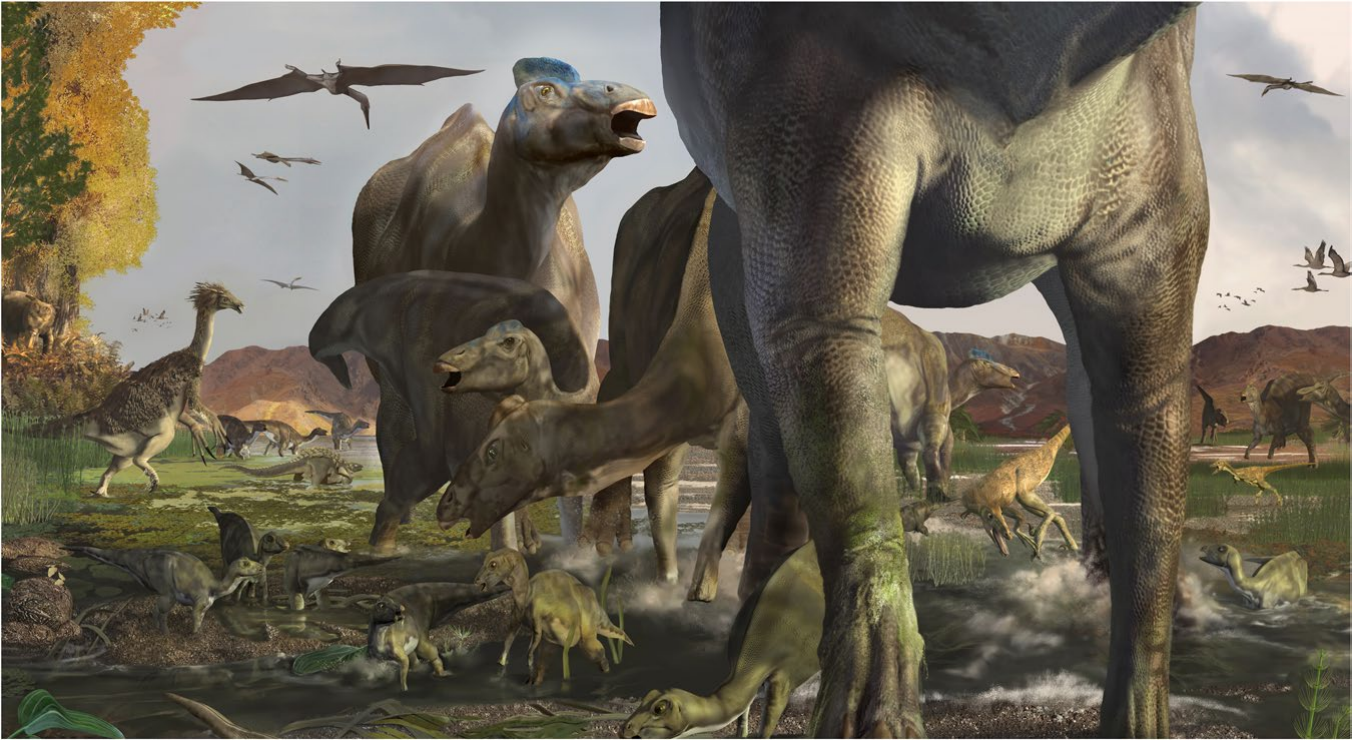
Life reconstruction of hadrosaur-therizinosaur co-occurrence based on tracks described in this report. Artwork by Karen Carr.

Within the dinosaur bone-bearing Prince Creek Formation of northern Alaska evidence indicates hadrosaurs preferred the more distal, wetter lower delta plain environments, in contrast to ceratopsid dinosaurs which seemed to prefer more proximal, slightly elevated, and drier upper coastal plain environments^78^. In addition to channel and splay deposits throughout the area, the presence of nymphaeaceous leaf fossils near the Big Creek track site shows that rivers and still-water ponds and lakes existed in this part of DENA at the time. The co-occurrence of tracks of hadrosaurids and therizinosaurs here, as well as body fossils of both taxa in the relatively wet paleoenvironment of the Nemegt Formation of Mongolia, seems more likely to be linked by an aspect of environment (i.e., abundance of water) rather than sophisticated multi-taxonomic herd behavior (Fig. 11).

While there is no definitive causal reason for this co-occurrence, the co-occurrence of hadrosaur tracks with therizinosaur tracks in the lower Cantwell Formation is decidedly unusual given the rich ichnologic record now known for this rock unit. Whether the animals interacted in a complex social manner or received mutually beneficial predatory avoidance for the combined herd is unresolved. The record of this co-occurrence in Beringia is important as it shows that there were subtle details and conditions within the ecosystem that facilitated a co-existence of these taxa at some level. The only other co-occurrence between these two clades now is found in central Asia. It suggests that there are important details of these ancient terrestrial ecosystems that remain to be determined, details that will help us understand the drivers for this faunal dynamic. It has long been recognized that Cretaceous Alaska represented the geographic gateway for faunal exchange between the Asian and North American, or at least Laramidian, continental landmasses. The ichnotaxonomic co-occurrence association reported here provides an intriguing scenario within the Cretaceous Beringian ecosystem, that an aspect of the continental ecosystem of central Asia made it into part of southern Alaska during the Latest Cretaceous.

## Methods

### Track 3D modeling

The surface morphology of each track was captured using photogrammetry utilizing molds made in the field^79–81^. Molds were used for photogrammetric modeling of the tracks because the appropriate photographs were not collected in the field at the time of discovery. Molds were created in the field using Smooth-On EquinoxTM 50 silicone putty. For one track specimen (DMNH 2013-08-04) an epoxy cast made from the mold was used for modeling. Photogrammetric models presented in Fig. 8 were generated from a series of digital photographs (compiled from sets of 56, 13, and 23 photos) using a Canon EOS Rebel T5i 18-megapixel camera (focal length 24–28 mm, resolution 5184 × 3456, pixel size 0.004384 mm). The photos were processed in Agisoft PhotoScan Pro (v.1.2.6) with all models having an error less than 0.5 pix. Post-processing of each mesh consisted of filling holes, trimming unnecessary edges, and adding an appropriate scale, and then exported as a binary STL file. Individual model data are available in Table S2. Original model files are provided in Supporting Information. The Z-axis of two models (DMNH 2013-08-06 and DMNH 2014-11-05) was inverted using Autodesk Meshmixer (v.3.0) in order to recreate the positive relief of the original tracks. Each mesh was aligned to a plane and colorized by Z-axis height in CloudCompare (v.2.7)^82,83^.

## Electronic supplementary material

Supplementary information
