# Supplementary material for: An unusual association of hadrosaur and therizinosaur tracks within Late Cretaceous rocks of Denali National Park, Alaska
Source: Sci Rep. 2018 Aug 3;8:11706. doi: 10.1038/s41598-018-30110-8 (PMC6076232; doi:10.1038/s41598-018-30110-8)
Supplement: Supplementary file 1 — Supplementary information [file 41598_2018_30110_MOESM1_ESM.pdf]

An unusual association of hadrosaur and therizinosaur tracks within Late Cretaceous rocks of Denali National Park, Alaska

Anthony R. Fiorillo, Paul J. McCarthy, Yoshitsugu Kobayashi, Carla S. Tomsich, Ronald S. Tykoski, Yuong-Nam Lee, Tomonori Tanaka, Christopher R. Noto

Supplemental Figure Legends

Supplement Fig. 1. Representative photographs and line drawings of additional therizinosaur tracks used in this study. Note the variation in track morphology within the sample. a-e correspond to field numbers 14AF7-15-3; 14AF7-16-2; 14AF7-16-4; 14AF7-17-6; 14AF7-17-7, respectively, in Supplemental Table 1.

Supplement Fig. 2: 3D mesh model for track DMNH 2013-08-04, produced in Agisoft PhotoScan Pro (v.1.2.6) in PDF format.

Supplement Fig. 3: 3D mesh model for track DMNH 2013-08-06, produced in Agisoft PhotoScan Pro (v.1.2.6) in PDF format.

Supplement Fig. 4: 3D mesh model for track DMNH 2014-11-05, produced in Agisoft PhotoScan Pro (v.1.2.6) in PDF format.

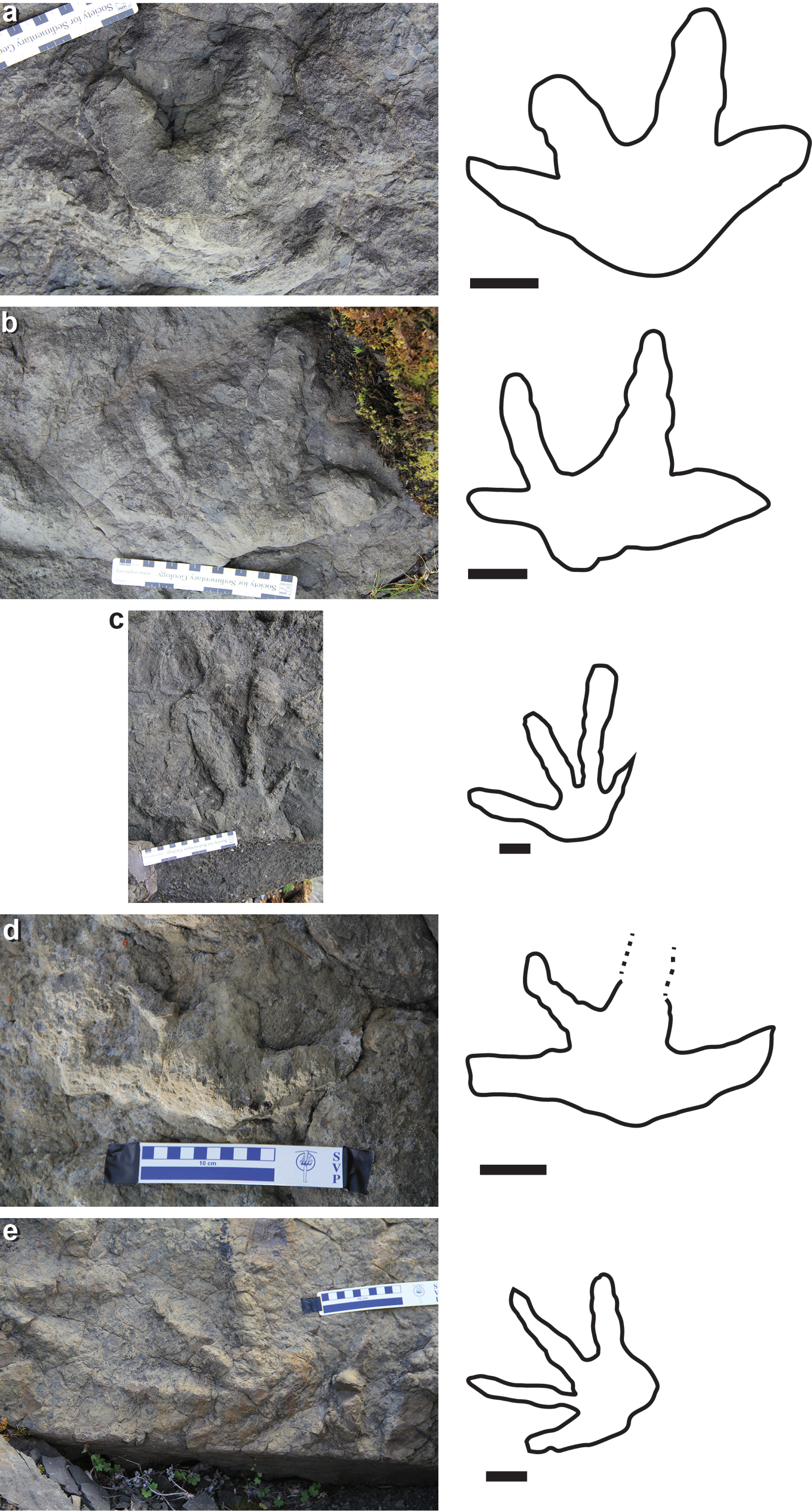
a
b
c
d
e
Society for Sedimentary Geology
10 cm
SVP

Generated with Agisoft PhotoScan

Generated with Agisoft PhotoScan

An unusual association of hadrosaur and therizinosaur tracks within Late Cretaceous rocks of Denali National Park, Alaska

Anthony R. Fiorillo1, Paul J. McCarthy, Yoshitsugu Kobayashi, Carla S. Tomsich, Ronald S. Tykoski, Yuong-Nam Lee, Tomonori Tanaka, Christopher R. Noto

Supplemental Table 1

Denali therizinosaur tracks

year found	field number	track number	length (cm)	width (cm)	mold*	specimen number	digit I-IV divarication angle
2013	13AF7-26-1		20	28	yes	DMNH 2013-08-04	119
	13AF7-26-3		24	23	yes	DMNH 2013-08-06	100
	13AF7-26-4		20	21			
2014	14AF7-13-3		13	18.5		DMNH 2014011-05	118
	14AF7-15-1	1	20.9	23.4			
		2	23.1	20.3	yes	DMNH 2010-07-01	118
		3	21.7	28.4			
	14AF7-15-2		19.8	35.2			
	14AF7-15-3		22	23			
	14AF7-15-4	1	20.8	28.3			125
		2	17.5	24.2			
	14AF7-15-5		16.1	22.3			
	14AF7-16-2		22.8	23.9			147
	14AF7-16-3		19.5	23.2			
	14AF7-16-4	1	24.2	26.9	yes		105
		2	23	26			
	14AF7-17-1	1	20.8	23.3			
		2	21.3	27.6			
	14AF7-17-2		19.5	24.5			
	14AF7-17-4	1	25.2	30.6			
		2	19.2	27.8			
	14AF7-17-5	1	14.5	18.5			
		2	17.2	20.3			
	14AF7-17-6		14.5	22.4			145
	14AF7-17-7	1	18.9	23.5			111
		2	20.4	23.8			
		3	18.4	24.2			
		4	18.5	23.5			
	14AF7-17-8	1	18.7	22.2			
		2	22.2	27.9			
	14AF7-18-1		18.2	19.4			

*unless indicated as 'yes,' all other tracks were measured in the field

An unusual association of hadrosaur and therizinosaur tracks within Late Cretaceous rocks of Denali National Park, Alaska

Anthony R. Fiorillo, Paul J. McCarthy, Yoshitsugu Kobayashi, Carla S. Tomsich, Ronald S. Tykoski, Yuong-Nam Lee, Tomonori Tanaka, Christopher R. Noto

Table S2. Mesh data for each specimen model.

Specimen	Number of photos used	Lens focal length (mm)	Average height from specimen (cm)	Number of triangles	Mesh area (cm^2)	Model Dimensions (cm)		
						Max width (X)	Max length (Y)	Max height (Z)
DMNH 2013-08-04	13	24	55	439,629	903.6	34.7	28.6	3.7
DMNH 2013-08-06	30	24	55	915,412	965.4	29.9	31.8	6.9
	26	28	35					
DMNH 2014-11-05	12	24	55	694,156	393.9	18.7	22.0	4.8
	11	27	35					
